# A Robot-Delivered Training Program to Improve Children’s Mental Health and Resilience in Dutch Primary Schools: Pilot Intervention Study

**DOI:** 10.2196/66797

**Published:** 2025-08-29

**Authors:** Anne Zijp, Jiska J Aardoom, Olivier A Blanson Henkemans, Sylvia van der Pal, Eline Vlasblom, Anke Versluis

**Affiliations:** 1 Department of Public Health and Primary Care, Leiden University Medical Center, Albinusdreef 2, Leiden, 2333 ZA, The Netherlands, 31 637402046; 2 Netherlands Organisation for Applied Scientific Research (TNO), Leiden, The Netherlands

**Keywords:** well-being, resilience, mental health, primary school children, robot-delivered intervention, robot, training program, child, mental, primary school, adulthood, depressive disorder, promotion, prevention, suicidal ideation, feasibility, acceptability, usability, pilot intervention, single-arm, suicide

## Abstract

**Background:**

Mental health problems often start at an early age and can persist into adulthood, leading to physical and mental health problems such as substance abuse, sleep problems, depressive disorders, and suicidal tendencies. Therefore, it is important to invest in the mental health of young people through, for example, initiatives focused on mental health promotion and prevention. The ePartners robot buddy offers children training modules focused on enhancing resilience and mental health, specifically targeting self-image and social skills and/or addressing unhelpful feelings and thinking patterns in children’s daily life situations.

**Objective:**

The study primarily aims to assess the feasibility, acceptability, and usability of the intervention according to the children and their teachers and secondarily aims to evaluate its potential effects on the mental well-being (general mental well-being, quality of life [QoL], and self-efficacy) of children.

**Methods:**

A single-arm, 6-week, pre-post pilot intervention study involving children and their teachers was conducted in 3 primary schools in the Netherlands. Outcomes were assessed using questionnaires. Primary outcomes were assessed postintervention and included feasibility and acceptability for teachers and acceptability and usability for children. Secondary outcomes included self-reported general mental well-being and self-efficacy and teacher-reported general mental well-being and were assessed at baseline and postintervention.

**Results:**

Data showed that the intervention was generally perceived as moderately feasible according to the Feasibility of Intervention Measure (mean 17.3, SD 2.6; on a scale from 4 to 20) and showed relatively high acceptability (mean 16.9, SD 3.8; on a scale from 4 to 20) according to teachers (n=7). Additional feasibility questions showed that teachers found it generally feasible to guide children who had few questions about using the robot. Feasibility was moderate due to limited time for integration, many content-related questions from children, and the substantial learning needed to select themes. Children (n=73) reported high perceived usability of the intervention (mean 15.2, SD 2.4; on a scale from 4 to 20). The perceived acceptability of the intervention by children was also relatively high, with a mean of 12.0 (SD 2.3) on a scale from 3 to 15. Teacher-reported QoL of children improved significantly from baseline (mean 36.0, SD 4.6) to postintervention (mean 37.2, SD 3.8; *t*_64_=2.77; *P*=.01); however, the children’s self-reported QoL did not significantly change over time. No significant changes in general mental well-being and self-efficacy scores were found.

**Conclusions:**

This study provides valuable insights into the feasibility, acceptability, and usability of a robot-delivered mental health–promoting intervention within a primary school setting. Further research is needed to fully understand its potential benefits and address existing limitations associated with the implementation of such interventions in the school setting.

## Introduction

Mental disorders represent a substantial global burden and rank among the top 10 leading causes of worldwide disease burden [1]. Concerning adolescents, nearly one-third (30.5%) of Dutch adolescents aged 12 to 16 years struggle with mental issues, with rates being higher for girls (39.8%) than for boys (21.5%) [2]. These mental health issues are already present in primary school (26.1%) and include hyperactivity/attention problems, emotional problems, problems with peers, and behavioral problems. A recent large-scale meta-analysis showed that almost two-thirds of mental disorders begin before the age of 25 years, and one-third start before the age of 14 years [3]. Experiencing mental health issues in youth often extends into adulthood, leading to physical and mental problems such as substance use, sleep issues, depressive disorders, and suicidal tendencies [4-6]. Therefore, it is important to invest in the mental health of young people through, for example, initiatives focused on mental health promotion and prevention.

Fostering resilience, defined as the ability to cope with stressful situations and successfully adapt to setbacks [7], can be considered a key factor in mental health promotion. Resilience encompasses the ability to bounce back from adversity, adapt positively to challenging circumstances, and maintain well-being in the face of stress or trauma. Resilience is a positive capacity that can be learned and, as such, can improve over time [8], making it a suitable target for mental health promotion initiatives. Indeed, resilience has been found to influence individuals’ health, well-being, and quality of life (QoL) [9-11]. Moreover, resilience-focused interventions have been found to improve mental health outcomes, such as depressive symptoms and general psychological distress, in children and adolescents [12]. The school environment provides a convenient setting for promoting mental health and fostering resilience in children. Previous studies on school-based mental health promotion and prevention programs have shown positive effects on children’s mental health. More specifically, a systematic review showed positive effects of resilience-focused mental health promotion programs in primary schools with regard to children’s ability to cope with daily stressors [13]. The reviewed programs were designed to enhance social and emotional skills with a strong emphasis on enhancing coping skills and strategies, such as mindfulness-based stress reduction techniques. These programs also aimed to improve social relationships, with the ultimate goal of improving mental well-being and safeguarding against future risks.

Social robots that can communicate and interact with people offer novel opportunities for mental health promotion. Social robots are increasingly being used in clinical and health settings to provide information, monitor health outcomes, and deliver psychosocial interventions [14-18]. Dawe et al [18] conducted a scoping review regarding social robots to help children in health care contexts. Fifty studies were identified, most of which were small feasibility studies. In these studies, the social robots were primarily used to provide companionship, support, and well-being through distraction and emotional support. They also served educational purposes (eg, providing information, demonstrating exercises, and delivering feedback) and provided assistance in stimulating social interactions and play among children. The results showed that social robots are generally readily and well accepted among children and that their use in health care is promising.

Building on research in this area, this pilot study examined a social robot–delivered mental health–promoting intervention in primary school. The intervention offers children training modules focused on enhancing resilience and mental health, specifically targeting self-image and social skills and/or addressing unhelpful feelings and thinking patterns in their daily life situations. The main aim of the pilot study was to investigate the feasibility, acceptability, and usability of the intervention according to the children and their teachers. A secondary aim was to evaluate its potential effects on the mental well-being of children.

## Methods

### Design and Population

A single-arm, 6-week, pre-post pilot intervention study was conducted. Data were collected from June to October 2023. The study population consisted of children and their teachers. Participants were recruited at 3 primary schools in the Netherlands: a special education school, a school in a low socioeconomic status neighborhood, and a school in a higher socioeconomic status neighborhood. In the Dutch primary school system, 1 teacher typically provides instruction to the same group of children throughout the entire week. Children remain in the same classroom, and the teacher covers most subjects. Grades 5, 6, and 7 generally include children aged 8‐10, 9‐11, and 10‐12 years, respectively. Children were eligible when they (1) attended grades 5, 6, or 7; (2) were able to speak, read, and understand the Dutch language; and (3) when their parents or guardians mastered the Dutch language to understand the information letter and informed consent form. Teachers were eligible to participate when they were teaching the participating children.

### Ethical Considerations

The Medical Ethical Committee of the Leiden University Medical Center in the Netherlands approved the protocol of the study (decision reference number WSC-2023‐05). According to the Medical Ethics Committee of Leiden University Medical Center, the study did not fall within the scope of the Dutch Medical Research Involving Human Subjects Act (23‐3026). All procedures were conducted in accordance with institutional guidelines and the Declaration of Helsinki. Informed consent was obtained from all parents of participating children before data collection. Participation was voluntary, and participants were free to withdraw at any time without consequence. Data were anonymized before analysis. No personal identifiers were retained, and access to the data was restricted to the research team members to ensure confidentiality. Participants did not receive individual compensation. As part of the study activities, the participating classes received an educational game as an incentive. No images or supplementary materials included in this paper allow for the identification of individual participants.

### Intervention

**Figure 1.**
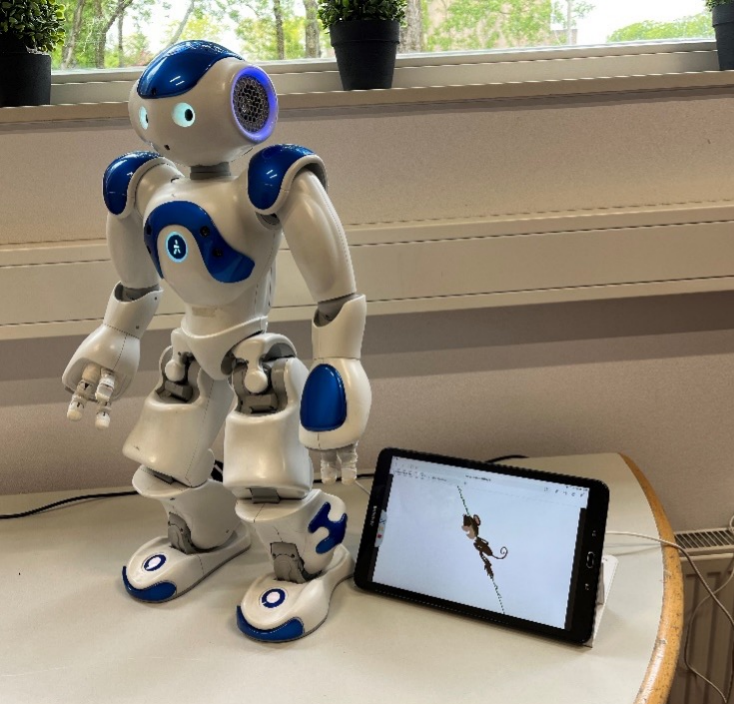
Intervention setup of the ePartners robot buddy with the tablet.

Each child individually followed a psychological training developed by Therapieland, a Dutch organization that offers evidence-based digital solutions for mental health care, focusing on one or two of the following themes: (1) self-image, (2) social skills, and (3) thinking, feeling, and doing. A short description of each theme can be found in Textbox 1, and a more elaborate description in Multimedia Appendix 1. The training was offered via a robot. The robot delivered the training by speaking text and asking questions to help children reflect on the module’s content, make connections to their own experiences, and create assignments for the upcoming week, as well as by showing educational videos via a tablet connected to the robot. An overview of the setup can be found in Figure 1. The child responded verbally to the robot or answered the robot’s question via the tablet. The robot provided reinforcing feedback on the process (eg, “Thank you for your answer”).

Textbox 1. **Three intervention themes.**

- Self-image: designed for children to develop a positive self-image. Recommended for children who are experiencing a negative self-image, anxiety, gloom, and/or stress.
- Social skills: designed for children to learn social skills techniques. Recommended for children who are experiencing insecurity and/or have limited social skills.
- Thinking, feeling, doing: designed for children to develop coping strategies for various situations. Recommended for children who are experiencing gloom, anxiety, negative self-image, and/or problems in emotion regulation.

**Figure 2.**
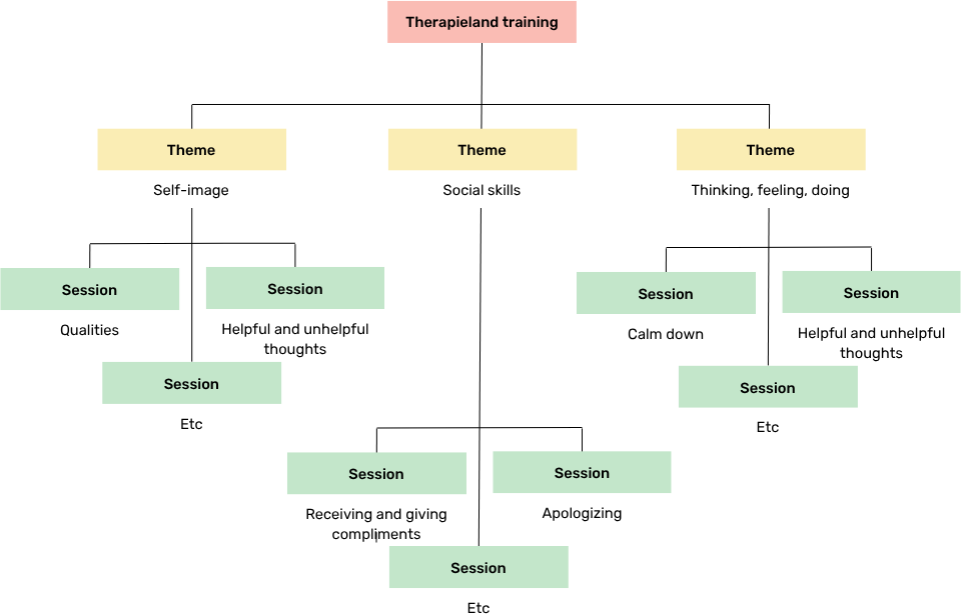
Overview of the themes of the psychological intervention ePartner robot buddy and examples of sessions within the themes.

The theme(s) the child worked on was based on the needs of the child as determined by either the parent(s), teacher, and/or researchers (see *Procedure* section). Within each theme, the teacher, child, and/or researchers decided which specific sessions were done (see *Procedure* section). An overview of the themes and an example of underlying sessions can be found in Figure 2. The child completed 1 session with the robot per week, for a total of 6 weeks. At the end of each session, the robot gave the child an exercise to complete at home and in the classroom (eg, compliment others this week and write down how it went). In the following session, the robot inquired whether the child practiced with the exercise and how they felt about it. The robot did not provide content-related feedback on the exercise but delivered process-related feedback, such as “yes it was a difficult exercise” or “let’s practice some more.”

### Procedure

Teachers of grades 5, 6, and 7 of the 3 participating schools received a digital information letter; researchers visited the school for a presentation of the robot and the research and invited teachers to participate. Interested teachers were asked to sign the informed consent form. Teachers were provided with an explanation of the intervention and procedure. Children were recruited through the participating teacher; they received an informative flyer. In addition, parents of the children received an official invitation through a school newsletter, a school app for parents, or on paper. This invitation included a QR code for the information letter, screening, and digital informed consent form. In case of questions, the parents were instructed to contact the teacher or the researchers.

After a child was included, the teacher determined which theme(s) was most suitable for the child based on an existing checklist that is periodically completed by the teacher as part of a student tracking system. This system, already implemented in the schools, is used for tracking student progress, results, and behaviors. The checklist provides scores on the child’s self-confidence, self-image, and social interactions, and these scores were used as a guideline for the selection of themes. The teacher then provided the selected suggestions for the theme(s) to the parent(s), and parents could respond in a face-to-face meeting (which would then be scheduled) or via email. If parents did not respond, the teacher decided on the theme(s). In some cases, teachers did not have time to select the theme(s), and the researcher chose the themes instead. By using this approach, the intervention could be tailored to the individual needs of each child. Next, the teacher completed the baseline KIDSCREEN-10 for the child (see *Measures* section).

At the first session, the child was asked by the researcher to complete the baseline questionnaire on general mental well-being and self-efficacy (see *Measures* section). Next, the researcher introduced the robot, and the robot also introduced itself using an automated introduction text. The researcher explained that each theme comprised different sessions, and the teacher and child together decided which sessions the child would complete in that week. There were a couple of exceptions where researchers chose the sessions because the teacher did not have sufficient time to do so.

The child received 1 session per week, each lasting up to 20 minutes, over a period of 6 weeks during school hours. Each session took place in a dedicated room where a robot and a tablet were prearranged, along with any required materials such as forms. To preserve the autonomous character of the intervention, the researcher remained present but out of sight. Upon arrival, the child was welcomed by the researcher and invited to sit in front of the robot. Once seated, the researcher stepped aside, and the robot independently initiated the session. During the session, the robot guided the child through various components, including educational animations or videos, reflective questions, and weekly assignments. The researcher intervened only in case of technical difficulties or if the child encountered confusion regarding the next steps. At the end of each session, an evaluation form was completed by the child to assess their experience. Sessions lasted approximately 15 minutes, consisting of a brief welcome and optional recap (3‐4 min), followed by an educational component (4‐5 min), a reflection and assignment phase (3‐4 min), and a short evaluation (1 min). Most children were assigned to the theme Self-image (n=32), followed by Feeling, thinking, doing (n=20), and Social skills (n=17), and some were assigned to sessions from both Feeling, thinking, doing and Self-image (n=4). At the end of the 6 weeks, the teacher completed the postintervention questionnaire on general mental well-being for the child, and the child completed the postintervention questionnaires assessing acceptability, usability, general mental well-being, and self-efficacy. After all the children in the school had completed the intervention, the corresponding teachers of that school completed the postintervention questionnaires assessing feasibility, acceptability, and additional measures. All participants completed pre- and postmeasurements.

### Measures

#### Feasibility

The intervention’s feasibility for teachers was assessed by the validated 4-item Feasibility of Intervention Measure [19]. Additionally, teachers were asked how feasible it was to determine the themes for the child, whether they had to learn a lot to determine the themes, and whether they had enough time to integrate the intervention in the school setting. All items were answered on a 5-point rating scale ranging from 1 (“completely disagree”) to 5 (“completely agree”). The feasibility of recruitment was assessed by the response rate, that is, the proportion of invited children and parents who provided informed consent.

#### Acceptability

For teachers, the intervention’s acceptability was assessed using the 4-item Acceptability of Intervention Measure [19]. Two additional questions were added to determine whether they (1) were satisfied with using the intervention in the school setting and (2) would use the intervention again. All items were answered on a 5-point Likert scale ranging from 1 (“completely disagree”) to 5 (“completely agree”).

In children, the intervention’s acceptability was assessed using 3 questions. It assessed whether they were satisfied with the robot, how helpful the robot was, and their willingness to use the robot again. The items were answered on a 5-point Smileyometer scale. The Smileyometer scale was presented to the children in a horizontal row with supporting words under the smiley faces [20]. Children were asked to pick 1 smiley face.

#### Usability

The intervention’s usability was assessed in children using 4 items. The items assessed whether the intervention was easy to use and could be carried out independently, as well as the difficulty level of the questions of the robot and the children’s ability to understand what the robot was saying. The items were answered on a 5-point Smileyometer scale.

#### General Mental Well-Being and Self-Efficacy as Reported by Children

Children’s self-reported general mental well-being and self-efficacy of children were assessed using 2 items from the validated Child Outcome Rating Scale [21]. Specifically, children were asked to answer the questions “How am I doing?” and “How am I doing at school?” The items were answered on a 10-point smiley face Likert scale.

Next, the Dutch version of KIDSCREEN-10 for children was used to assess children’s self-reported QoL [22]. The 10 questions ask about the child’s experiences over the past week, with an example item being “Have you felt fit and well?” One additional question aimed to assess the child’s general health (ie, “In general, how would you say your health is?”). All questions were answered on a 5-point Likert scale. Higher scores indicate a better QoL.

Additionally, the 8-item Self-Efficacy Questionnaire for Children was administered [23]. An example item is “How well can you become friends with other children?” The items were answered on a 5-point smiley face Likert scale. Higher scores indicate a better self-efficacy.

#### Children’s General Mental Well-Being as Reported by Teachers

To complement the children’s self-reported QoL, proxy reports from the teachers were used [24]. Specifically, children’s mental health was assessed with the Dutch KIDSCREEN-10 questionnaire completed by their teacher. The teacher questionnaire included the same questions as the child version, but from the teacher’s perspective (eg, “Has the child felt fit and well?”).

#### Additional Measures

Sociodemographic characteristics, including age and gender, were assessed in children at baseline and postintervention in teachers. General experiences of teachers with the intervention in the school setting were assessed by 3 open-ended questions administered at postintervention (ie, How did you experience the training sessions with the robot in the school setting? How do you think the children experienced the training with the robot? Would you like to say anything else about the training with the robot in the school setting?). These questions aimed to get a better understanding of their experiences.

In addition, before conducting each session, children were asked to respond to 5 questions regarding their experiences with the latest session: what they thought of last week’s session (pleasant/neutral/unpleasant), how they felt last week’s session went (good/neutral/bad), what they thought of the level of difficulty (too difficult/somewhat difficult/just right/somewhat easy/too easy), whether they did try practicing with it at home (yes/no), and how important they found it to learn the task with the robot from last week (yes/no).

### Analyses

Descriptive analyses (eg, mean [SD]*,* N, and percentages) were used to summarize the results regarding sociodemographic characteristics, feasibility, acceptability, usability, mental well-being (both children’s self-report and teacher-reported), and self-efficacy. To identify potential change over time, the total scores on the Child Outcome Rating Scale, KIDSCREEN-10, and 8-item Self-Efficacy Questionnaire for Children were statistically compared between pre- and postintervention using paired samples (2-tailed) *t* tests. Data were only analyzed in case a child completed 5 or more sessions. The analyses were performed using SPSS (version 25; IBM Corp) [25]. The free-text answers to the open-ended questions were summarized.

## Results

### Primary Research Question: Feasibility, Acceptability, and Usability of the Robot

#### Feasibility and Acceptability According to Teachers

Seven teachers were included and completed the postintervention questionnaires. The mean age was 41.3 years (range 33‐63 years); of 7 teachers, 3 were male and 4 were female. Table 1 shows the results regarding feasibility and acceptability. The intervention’s feasibility was scored high. The additional feasibility questions showed that teachers found it generally feasible to guide the children and set themes for the children. Furthermore, children did not ask many questions about how to use the robot. Feasibility scores were moderate in terms of having enough time to integrate the intervention into the school setting. According to the teachers, the feasibility of the intervention was considered to be limited by children asking many questions about the content of the sessions and due to the considerable amount of learning required to be able to determine the themes for the child.

The general acceptability of the intervention was reasonably high. Furthermore, teachers generally reported being satisfied with the use of the intervention in the school setting and that they would like to use the intervention more often.

Three of the 7 teachers from 2 schools completed the open-ended questions, of whom 2 from the same school experienced the intervention positively. They indicated that it was a well-organized intervention and that children enjoyed working with the robot. One teacher was less positive, highlighting the amount of administration and the fact that the children did not give positive feedback on the intervention. When asked if they had anything else to say about the intervention in the school setting, one mentioned that the possibilities of the intervention were not clear for one teacher, whereas another teacher from another school felt that it could be managed well within the school setting. One teacher suggested areas for improvement:

*I think a robot should communicate with every pupil, asking a student a predetermined number of questions to prompt an internal dialogue in student. This will increase the “coaching” effect of the robot.*

**Table 1.** Perceived feasibility and acceptability of the intervention ePartners robot buddy according to teachers.

			Mean (SD)	
FIM^a^ score (range 4‐20)			17.3 (2.6)	
Additional feasibility questions (score; range 1‐5)				
Guiding the child/children was feasible			4.1 (0.4)	
It is feasible to set themes for the children			3.9 (0.7)	
It was feasible to define the themes for the children together with the parents			3.6 (1.5)	
I feel I have enough time to integrate the intervention into the school setting			3.3 (1.4)	
To determine the themes, I had to learn a lot^b^			2.8 (1.6)	
The child/children asked many questions about the content of the sessions^b^			2.5 (2.0)	
The child/children asked many questions about using the robot^b^			1.5 (0.8)	
AIM^c^ score (range 4‐20)			16.9 (3.8)	
Additional acceptability questions (score; range 1‐5)				
I am satisfied with the use of the intervention in the school setting			4.1 (0.7)	
I would like to use the intervention more in the school setting			4.0 (0.8)	

a FIM: Feasibility of Intervention Measure.

b A higher score means lower feasibility.

c AIM: Acceptability of Intervention Measure.

#### Acceptability and Usability According to Pupils

A total of 73 children were included and completed the pre- and postintervention questionnaires. The mean age was 9.9 years (SD 1.1 years, range 8‐12), and the ratio of boys versus girls was nearly equal (41/73, 55.4% boys). Children reported high perceived usability of the intervention (mean 15.2, SD 2.4). The perceived acceptability of the intervention was also scored relatively high with a mean of 12.0 (SD 2.3).

The additional questions administered before each session indicate that most children found the sessions pleasant, reported that the session went good, and perceived the difficulty level as just right. On most occasions, a small majority of children indicated that they had not practiced at home with the robot. However, the children expressed the importance of engaging in the session with the robot (Table 2).

**Table 2.** Children’s experiences with the ePartners robot buddy sessions.

Question and answer options		n (%)	
What did you think of last week’s session?			
Pleasant		163 (75.8)	
Neutral		26 (12.1)	
Unpleasant		8 (3.7)	
Missing		18 (8.4)	
How did last week’s session go?			
Good		161 (74.9)	
Neutral		31 (14.4)	
Bad		5 (2.3)	
Missing		18 (8.4)	
Did you find the session difficult/easy?			
Too difficult		0 (0)	
Somewhat difficult		30 (14.0)	
Just right		103 (47.9)	
Somewhat easy		39 (18.1)	
Too easy		25 (11.6)	
Missing		18 (8.4)	
Did you try practicing with it at home?			
Yes		83 (38.6)	
No		112 (52.1)	
Missing		20 (9.3)	
Did you find it important to learn the session from last week with the robot?			
Yes		185 (86.0)	
No		12 (5.6)	
Missing		18 (8.4)	

### Secondary Research Question: Preliminary Effects of the Robot

The results regarding mental well-being can be found in Table 3. The teacher-reported QoL of the children was significantly improved at postintervention as compared to baseline with a small to medium effect size. However, the children’s self-reported QoL did not significantly change over time and had a small effect size. In addition, no significant changes in general mental well-being and self-efficacy scores of children were found.

**Table 3.** Data of secondary outcome measures at baseline and postintervention, along with the results of paired *t* tests of the preliminary effects of the ePartners robot buddy.

	Baseline, mean (SD)	Postintervention, mean (SD)	*t* test		Cohen *d*
			*t* (*df*)	*P* value	
General mental well-being					
How are you doing?	8.8 (1.6)	8.8 (1.5)	0 (64)	>.99	0
How are you doing at school?	8.3 (1.7)	8.6 (1.7)	1.10 (64)	.28	0.14
Quality of life					
Teacher-reported KIDSCREEN-10	36.0 (4.6)	37.2 (3.8)	2.77 (64)	.01	0.34
Children’s self-reported KIDSCREEN-10	37.8 (3.5)	38.5 (3.6)	1.56 (61)	.10	0.21
Self-efficacy					
Self-reported SEQ-C^a^	28.9 (4.8)	29.8 (5.6)	1.62 (57)	.11	0.21

a SEQ-C: 8-item Self-Efficacy Questionnaire for Children.

## Discussion

### Principal Findings

The pilot study primarily investigated the feasibility, acceptability, and usability of a robot-delivered mental health–promoting intervention within a primary school setting. The results showed that the intervention was generally perceived as moderately feasible and acceptable according to both teachers and children, although several challenges were identified with the implementation and integration of the intervention into the school setting. More specifically, teachers were not always able to find the time to select the most suitable themes for the children or to consult with the child and parents about which sessions within these themes he/she would be best suited to attend. Furthermore, addressing children’s questions regarding the content of the intervention sessions, as well as the amount of learning required to determine suitable intervention themes for the children, required a substantial time investment by the teachers.

The identified challenges with implementation and integration underscore the importance of considering practical barriers when introducing new programs into educational environments, such as primary schools. Both on a national and international scale, there is a shortage of teachers, leading to increased workload and corresponding limited resources [26,27]. Teachers’ limited time availability for tasks related to adequate implementation of mental health–promoting interventions highlights the need for streamlined and efficient intervention protocols that minimize the burden on educators. Possibly, the social domain could provide support through training and coaching or coordinating resources, thereby supporting educational institutions and their staff. Another way to address such implementation challenges within school contexts is to actively engage teachers and school personnel in the development and implementation process of such interventions to ensure that the intervention aligns with the school’s curriculum and resources. The significant time investment required from teachers to manage children’s inquiries about the robot and the intervention content furthermore highlights the importance of carefully designing such interventions in co-creation with the target population, so that such programs meet their needs and abilities effectively. Another possibility is to enable the robot to select and conduct tasks with the child without the assistance of a human facilitator. Addressing these challenges is essential to ensure the successful integration and sustainability of mental health interventions within school settings, ultimately maximizing their potential benefits for children’s well-being.

Regarding the preliminary effects of the intervention on children’s mental well-being, the findings suggest that while the intervention may have a positive impact on teacher-perceived aspects of children’s well-being, its effects on self-reported measures and broader mental health outcomes may be limited or require further investigation. The latter has been highlighted as a future research topic as well in recent reviews on robot-delivered interventions in the field of mental health [14,15,18]. Furthermore, it should be noted that there was no information available about which specific sessions the children attended within the intervention themes. It is possible that different sessions could have different impacts; this aspect warrants consideration for future research. In addition to studying the effects of mental health–promoting interventions delivered by robots, a more thorough investigation of specific needs of children and teachers is needed, for example, using a qualitative study design exploring end-user needs in more detail.

### Strengths and Limitations

A strength of this study was its multischool design. However, the small sample size and potential selection bias due to children being selected for the intervention by their teachers limit the generalizability of the findings. In addition, the fact that 2 of the 3 schools started the intervention after the summer holidays may have caused a bias when teachers completed questionnaires about the child, as their knowledge about the children’s well-being may have been limited at that time and improved over the duration of the pilot.

### Conclusions

This study provides valuable insights into the feasibility, acceptability, and usability of a robot-delivered mental health–promoting intervention within a primary school setting. However, further research is needed to fully understand its potential benefits and address existing limitations in terms of the implementation of such interventions in the primary school setting. In addition, the burden on teachers is a crucial factor in the feasibility of the intervention within the primary school setting. Therefore, the robot must be intelligent and personalized, enabling students to use it independently.

## Supplementary material

10.2196/66797 Multimedia Appendix 1 Description of the intervention themes.

## Abbreviations

QoL: quality of life
