# Supplementary material for: A Robot-Delivered Training Program to Improve Children’s Mental Health and Resilience in Dutch Primary Schools: Pilot Intervention Study
Source: JMIR Form Res. 2025 Aug 29;9:e66797. doi: 10.2196/66797 (PMC12396791; doi:10.2196/66797)
Supplement: Multimedia Appendix 1 [file formative-v9-e66797-s001.docx]

**Appendix 1. Description of the intervention themes.**

Theme 1: Self-image

This training is designed to help children develop a positive self-image and is recommended for children experiencing a negative self-image, anxiety, gloom and/or stress. In this training, the child gains self-knowledge about one’s skills and personal qualities, and will learn more about helpful and unhelpful thoughts. Within this theme, there are multiple sessions available (e.g. qualities, helpful and unhelpful thoughts).

The child sets goals, including gaining insight into one's own qualities; learning to identify and formulate helpful thoughts and distinguish them from unhelpful thoughts; learning to deal with positive emotions, receiving compliments and being proud of oneself; learning to deal with negative emotions, turning negative emotions into positive emotions and responding to difficult moments.

Theme 2: Social skills

This training is designed for children to learn social skills techniques. This training is recommended for children who are experiencing insecurity and/or have limited social skills. Within this theme, there are multiple sessions available, such as receiving and giving compliments, and apologizing. The sessions cover various skills needed to have a conversation, such as how to get to know another, listening, asking questions and learn to say sorry. For example, the participant is given information and exercises on recognizing and dealing with emotions or playing together, and how to cope with bullying.

Theme 3: Thinking, feeling, doing

This training is designed for children to develop coping strategies for various situations. This training is recommended for children who are experiencing gloom, anxiety, negative self-image, problems in emotion regulation. Within this theme, there are multiple session available (e.g. helpful and unhelpful thoughts, and calm down). The training starts with animated psycho-education about thoughts, feelings and behaviour. These are animations of animals; a lion, an owl, a giraffe and a turtle. These are all different animals with different thinking patterns. G-schemes will be used to list an event and the thought, feeling and behaviour that follows (*in Dutch: gebeurtenis, gedachte, gevoel, gedrag*). Helpful thoughts and behaviours will be discussed.

An example of a snippet of text the robot speaks in a ‘helping thoughts’ session is:

*‘I do like to teach you how to help yourself feel better, even at unpleasant times. This lesson is about helpful and non-helpful thoughts.’*

*‘In a day, or in a week, nice things happen, and sometimes unpleasant things happen. In all of these situations, you have thoughts. Some thoughts make you happy or give you confidence. These are called helpful thoughts. But other thoughts might make you angry or sad. Those are called unhelpful thoughts, or thinking errors.”*

*‘I'm going to ask you a question to see if you know which thoughts are helpful or not. How about the following thought? "I never do anything right." Is that helpful? say YES or NO or tap on the screen for yes or no*.’
